# Ethyl 1’-[1-(4-methoxyphenyl)-3-phenoxy-4-phenylazetidin-1-yl]-1,3-dioxo-2′,3′,5′,6′,7′,7a′-hexahydroindan-2-spiro-3′-1′*H*-pyrrolizine-2′-carboxylate

**DOI:** 10.1107/S1600536808026913

**Published:** 2008-08-30

**Authors:** E. Theboral Sugi Kamala, S. Nirmala, L. Sudha, N. Arumugam, R. Raghunathan

**Affiliations:** a Department of Physics, Easwari Engineering College, Ramapuram, Chennai 600 089, India; b Department of Physics, SRM University, Ramapuram Campus, Chennai 600 089, India; c Department of Organic Chemistry, University of Madras, Guindy Campus, Chennai 600 025, India.

## Abstract

In the title compound, C_34_H_32_N_2_O_7_, the methyl group and methylene H atoms of the ethoxycarbonyl substituent are disordered over two positions with site occupancy factors for the major and minor conformers of 0.594 (8) and 0.406 (8), respectively. The unsubstituted ring of the pyrrolizine ring system exhibits a twist conformation, the other an envelope conformation. In the crystal structure, mol­ecules are linked through C—H⋯O hydrogen bonds; intramolecular C—H⋯O interactions are also observed.

## Related literature

For related literature, see: Allen *et al.* (1987 ▶); Alonso *et al.* (2002 ▶); Aoyama *et al.* (2001 ▶); Chande *et al.* (2005 ▶); Cremer & Pople (1975 ▶); Escolano & Jones (2000 ▶); Halve *et al.* (2007 ▶); Kamala *et al.* (2008 ▶); Nardelli (1983 ▶); Pinna *et al.* (2002 ▶); Poornachandran & Raghunathan (2006 ▶); Raj & Raghunathan (2001 ▶); Raj *et al.* (2003 ▶).
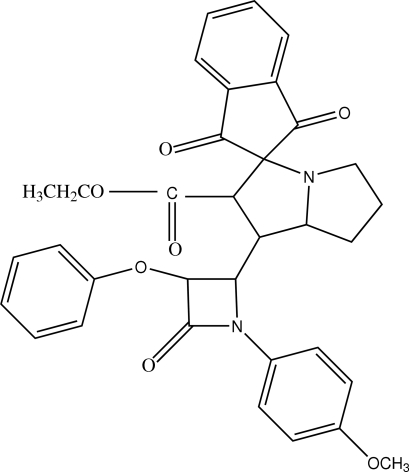


## Experimental

### 

#### Crystal data

C_34_H_32_N_2_O_7_
*M* *_r_* = 580.62
Monoclinic, 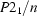

*a* = 12.4776 (7) Å
*b* = 12.4946 (6) Å
*c* = 19.4958 (11) Å
β = 106.013 (3)°
*V* = 2921.5 (3) Å^3^
*Z* = 4
Mo *K*α radiation
μ = 0.09 mm^−1^
*T* = 293 (2) K
0.30 × 0.20 × 0.20 mm

#### Data collection

Bruker Kappa APEXII diffractometer
Absorption correction: multi-scan (Blessing, 1995 ▶) *T*_min_ = 0.973, *T*_max_ = 0.982
66713 measured reflections
7446 independent reflections
4654 reflections with *I* > 2σ(*I*)
*R*_int_ = 0.037

#### Refinement

*R*[*F* ^2^ > 2σ(*F* ^2^)] = 0.057
*wR*(*F* ^2^) = 0.225
*S* = 0.92
7446 reflections
395 parameters
H-atom parameters constrained
Δρ_max_ = 0.70 e Å^−3^
Δρ_min_ = −0.25 e Å^−3^

### 

Data collection: *APEX2* (Bruker, 2004 ▶); cell refinement: *APEX2* and *SAINT* (Bruker, 2004 ▶); data reduction: *SAINT* and *XPREP* (Bruker, 2004 ▶); program(s) used to solve structure: *SHELXS86* (Sheldrick, 2008 ▶); program(s) used to refine structure: *SHELXL97* (Sheldrick, 2008 ▶); molecular graphics: *ORTEP-3* (Farrugia, 1997 ▶); software used to prepare material for publication: *PLATON* (Spek, 2003 ▶).

## Supplementary Material

Crystal structure: contains datablocks I, global. DOI: 10.1107/S1600536808026913/bt2772sup1.cif

Structure factors: contains datablocks I. DOI: 10.1107/S1600536808026913/bt2772Isup2.hkl

Additional supplementary materials:  crystallographic information; 3D view; checkCIF report

## Figures and Tables

**Table 1:** Hydrogen-bond geometry (Å, °)

*D*—H⋯*A*	*D*—H	H⋯*A*	*D*⋯*A*	*D*—H⋯*A*
C2—H2⋯O6^i^	0.98	2.45	3.151 (3)	129
C26—H26⋯O1^ii^	0.93	2.50	3.390 (4)	160
C8—H8*A*⋯O6	0.97	2.50	3.166 (4)	126
C10—H10⋯O2	0.98	2.24	2.987 (3)	132
C29—H29⋯O1	0.93	2.40	3.024 (3)	125

Symmetry codes: (i) 
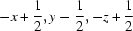
; (ii) 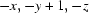.

**Fig. 1.**
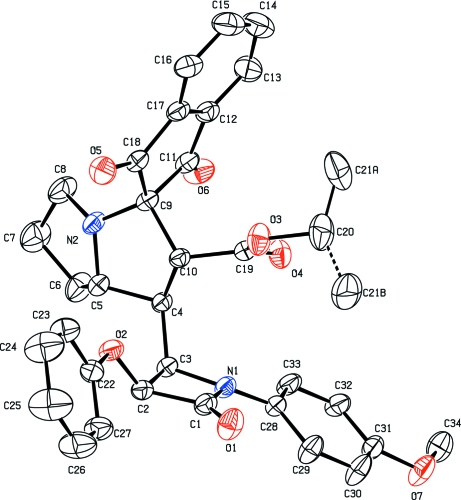
The molecular structure of (I) with 30% probability displacement ellipsoids.

**Fig. 2.**
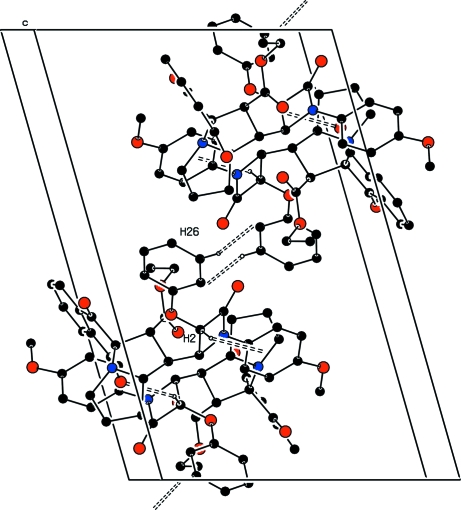
The packing of the molecules viewed along b axis.

## supplementary crystallographic information

### Comment

Pyrrolidine derivatives are widely used as organic catalysts and also serve as important structural units in biologically active molecules. Pyrrolidine derivatives, apart from displaying important biological activities (Pinna *et al.*, 2002; Escolano & Jones, 2000), are present in natural products such as cephalotoxin, kainic acid, domoic acid and quinocarcin. The cycloaddition of azomethine ylides to dipolarophiles with exocyclic double bonds affords spiro-pyrrolidines (Raj & Raghunathan, 2001; Poornachandran & Raghunathan, 2006), which display important biological activities (Raj *et al.*, 2003). In view of their very good antimycobacterial activity,synthesis of spiro compounds has drawn considerable attention from chemists (Chande *et al.*, 2005). The azetidinone ring system is the common structural feature of a number of broad spectrum β-lactam antibiotics (Halve *et al.*, 2007) and also possesses other pharmalogical properties (Aoyama *et al.*, 2001). They are precursors of α,α-disubstituted β-amino acids (Alonso *et al.*,2002).

Fig. 1 shows the *ORTEP* (Farrugia,  1997) plot of compound (I). Bond lengths and angles are comparable with other reported values (Allen *et al.*,1987; Kamala *et al.*,2008). The atom C21 is disordered.

In the molecule the five membered rings N2/C5/C4/C10/C9 and C9/C11/C12/C17/C18 exhibit *envelope* conformation with envelopes on C10 and C9 respectively with the assymetry parameters (Nardelli,1983) ΔC_s_(C10)/C(9) = 2.3 (2)/1.2 (2) and with the puckering parameters (Cremer and Pople,1975) q2 = 0.368 (2) Å/ 0.113 (2)Å and φ2 =104.4 (3)° / 184.2 (12)°. The pyrrolidine ring N2/C5—C8 exhibits *twist* conformation with assymetry parameter ΔC_2_ (N2) =3.1 (3), and with the puckering parameters q2 = 0.365 (3) Å, φ2 = 272.4 (4)°.

The sum of bond angles around N1 [354.96°] and that around atom N2 [343.25°] indicate *sp*^2^ hybridizations. The azetidinone ring is almost perpendicular to the pyrrolidine ring N2/C4/C5/C9/C10 making a dihedral angle of 88.70 (8)°. The other pyrrolidine ring N2/C5—C8 makes a dihedral angle of 80.15 (6)° with the central β lactam moiety while that of the phenyl ring is 60.19 (9)°.

In the crystal packing, atoms O1 and O6 are involved in intermolecular C - H···O interactions and atoms O1 and O2 are involved in intramolecular C - H···O interactions.

### Experimental

To a solution of ninhydrin (1 mmol), proline (1 mmol) in dry acetonitrile (10 ml) was added β-azetidinyl crotonic ester (1 mmol) under nitrogen atmosphere. The solution was refluxed for 2–3 h. After completion of reaction the solvent was distilled off under reduced pressure. The crude product was purified by column chromatography using hexane: ethylacetate (8:2) as eluent. The product was crystallized by ethylacetate.

### Refinement

Atom C21 is disordered over two positions (C21A/C21B), with refined occupancies of 0.594 (8) and 0.406 (8). H atoms were placed in idealized positions and allowed to ride on their parent atoms, with C–H = 0.93 or 0.96 Å and *U*_iso_(H)= 1.2–1.5*U*_eq_(C).

### Crystal data

**Table:**

C_34_H_32_N_2_O_7_	*F*_000_ = 1224
*M**_r_* = 580.62	*D*_x_ = 1.320 Mg m^−^^3^
Monoclinic, *P*2_1_/*n*	Mo *K*α radiation λ = 0.71073 Å
Hall symbol: -P 2yn	Cell parameters from 66713 reflections
*a* = 12.4776 (7) Å	θ = 2.0–28.7º
*b* = 12.4946 (6) Å	µ = 0.09 mm^−^^1^
*c* = 19.4958 (11) Å	*T* = 293 (2) K
β = 106.013 (3)º	Prism, colourless
*V* = 2921.5 (3) Å^3^	0.30 × 0.20 × 0.20 mm
*Z* = 4	

### Data collection

**Table:**

Bruker Kappa APEXII diffractometer	7446 independent reflections
Radiation source: fine-focus sealed tube	4654 reflections with *I* > 2σ(*I*)
Monochromator: graphite	*R*_int_ = 0.037
*T* = 293(2) K	θ_max_ = 28.7º
ω and φ scan	θ_min_ = 2.0º
Absorption correction: multi-scan(Blessing, 1995)	*h* = −16→16
*T*_min_ = 0.973, *T*_max_ = 0.982	*k* = −16→16
66713 measured reflections	*l* = −26→26

### Refinement

**Table:**

Refinement on *F*^2^	Secondary atom site location: difference Fourier map
Least-squares matrix: full	Hydrogen site location: inferred from neighbouring sites
*R*[*F*^2^ > 2σ(*F*^2^)] = 0.057	H-atom parameters constrained
*wR*(*F*^2^) = 0.225	*w* = 1/[σ^2^(*F*_o_^2^) + (0.1376*P*)^2^ + 1.4238*P*] where *P* = (*F*_o_^2^ + 2*F*_c_^2^)/3
*S* = 0.92	(Δ/σ)_max_ < 0.001
7446 reflections	Δρ_max_ = 0.70 e Å^−^^3^
395 parameters	Δρ_min_ = −0.25 e Å^−^^3^
Primary atom site location: structure-invariant direct methods	Extinction correction: none

### Special details

**Table:**

Geometry. All e.s.d.'s (except the e.s.d. in the dihedral angle between two l.s. planes) are estimated using the full covariance matrix. The cell e.s.d.'s are taken into account individually in the estimation of e.s.d.'s in distances, angles and torsion angles; correlations between e.s.d.'s in cell parameters are only used when they are defined by crystal symmetry. An approximate (isotropic) treatment of cell e.s.d.'s is used for estimating e.s.d.'s involving l.s. planes.
Refinement. Refinement of *F*^2^ against ALL reflections. The weighted *R*-factor *wR* and goodness of fit *S* are based on *F*^2^, conventional *R*-factors *R* are based on *F*, with *F* set to zero for negative *F*^2^. The threshold expression of *F*^2^ > σ(*F*^2^) is used only for calculating *R*-factors(gt) *etc*. and is not relevant to the choice of reflections for refinement. *R*-factors based on *F*^2^ are statistically about twice as large as those based on *F*, and *R*- factors based on ALL data will be even larger.

### Fractional atomic coordinates and isotropic or equivalent isotropic displacement parameters (Å^2^)

**Table:**

	*x*	*y*	*z*	*U*_iso_*/*U*_eq_	Occ. (<1)
C1	0.10567 (17)	0.74081 (19)	0.12572 (12)	0.0482 (5)	
C2	0.20897 (17)	0.67935 (17)	0.16633 (12)	0.0457 (5)	
H2	0.1884	0.6121	0.1852	0.055*	
C3	0.22628 (15)	0.76987 (17)	0.22302 (11)	0.0414 (4)	
H3	0.2171	0.7419	0.2680	0.050*	
C4	0.32801 (15)	0.84224 (16)	0.23708 (10)	0.0386 (4)	
H4	0.3175	0.9013	0.2676	0.046*	
C5	0.43512 (16)	0.78329 (19)	0.27532 (11)	0.0456 (5)	
H5	0.4292	0.7084	0.2598	0.055*	
C6	0.4746 (2)	0.7876 (3)	0.35635 (13)	0.0686 (8)	
H6A	0.4487	0.7259	0.3775	0.082*	
H6B	0.4493	0.8524	0.3745	0.082*	
C7	0.6006 (3)	0.7866 (3)	0.37091 (16)	0.0828 (9)	
H7A	0.6364	0.8171	0.4173	0.099*	
H7B	0.6280	0.7143	0.3691	0.099*	
C8	0.6216 (2)	0.8539 (3)	0.31244 (15)	0.0715 (8)	
H8A	0.6283	0.9288	0.3259	0.086*	
H8B	0.6893	0.8315	0.3013	0.086*	
C9	0.48118 (15)	0.91979 (17)	0.20062 (11)	0.0413 (4)	
C10	0.35503 (15)	0.88984 (16)	0.17155 (10)	0.0383 (4)	
H10	0.3473	0.8337	0.1354	0.046*	
C11	0.49581 (16)	1.03640 (19)	0.22699 (12)	0.0470 (5)	
C12	0.53156 (17)	1.10040 (19)	0.17360 (12)	0.0493 (5)	
C13	0.5449 (2)	1.2101 (2)	0.17030 (17)	0.0686 (7)	
H13	0.5286	1.2553	0.2040	0.082*	
C14	0.5828 (3)	1.2501 (3)	0.11582 (19)	0.0835 (9)	
H14	0.5923	1.3235	0.1126	0.100*	
C15	0.6071 (3)	1.1836 (3)	0.06565 (17)	0.0780 (9)	
H15	0.6333	1.2131	0.0296	0.094*	
C16	0.5934 (2)	1.0753 (2)	0.06794 (14)	0.0607 (6)	
H16	0.6095	1.0307	0.0339	0.073*	
C17	0.55463 (16)	1.03373 (19)	0.12275 (11)	0.0468 (5)	
C18	0.53427 (16)	0.92134 (18)	0.13783 (11)	0.0443 (5)	
C19	0.28198 (16)	0.98272 (18)	0.13906 (11)	0.0425 (4)	
C20	0.1937 (3)	1.0703 (3)	0.03029 (16)	0.0873 (10)	
H20A	0.1358	1.0866	0.0532	0.105*	0.594 (8)
H20B	0.1582	1.0475	−0.0182	0.105*	0.594 (8)
H20C	0.2032	1.1346	0.0593	0.105*	0.406 (8)
H20D	0.2132	1.0867	−0.0134	0.105*	0.406 (8)
C21A	0.2620 (3)	1.1658 (3)	0.02972 (16)	0.107 (3)	0.594 (8)
H21A	0.2156	1.2225	0.0044	0.161*	0.594 (8)
H21B	0.3185	1.1494	0.0065	0.161*	0.594 (8)
H21C	0.2966	1.1881	0.0779	0.161*	0.594 (8)
C21B	0.0759 (8)	1.0331 (10)	0.0133 (6)	0.122 (5)	0.406 (8)
H21D	0.0275	1.0881	−0.0123	0.184*	0.406 (8)
H21E	0.0571	1.0175	0.0568	0.184*	0.406 (8)
H21F	0.0672	0.9697	−0.0155	0.184*	0.406 (8)
C22	0.27365 (19)	0.58488 (18)	0.08017 (12)	0.0491 (5)	
C23	0.3552 (2)	0.5747 (2)	0.04537 (14)	0.0640 (7)	
H23	0.4182	0.6181	0.0576	0.077*	
C24	0.3422 (3)	0.4997 (4)	−0.00750 (19)	0.0970 (12)	
H24	0.3968	0.4920	−0.0314	0.116*	
C25	0.2488 (4)	0.4355 (4)	−0.0256 (2)	0.1091 (14)	
H25	0.2407	0.3848	−0.0616	0.131*	
C26	0.1680 (3)	0.4462 (3)	0.00915 (19)	0.0888 (10)	
H26	0.1052	0.4025	−0.0030	0.107*	
C27	0.1798 (2)	0.5210 (2)	0.06166 (15)	0.0627 (6)	
H27	0.1246	0.5290	0.0850	0.075*	
C28	0.03902 (16)	0.87881 (18)	0.19790 (11)	0.0432 (5)	
C29	−0.07248 (19)	0.8653 (2)	0.16113 (14)	0.0612 (7)	
H29	−0.0927	0.8169	0.1235	0.073*	
C30	−0.1525 (2)	0.9233 (3)	0.18030 (15)	0.0716 (8)	
H30	−0.2271	0.9139	0.1557	0.086*	
C31	−0.12380 (17)	0.9960 (2)	0.23592 (13)	0.0521 (5)	
C32	−0.01383 (18)	1.0081 (2)	0.27267 (13)	0.0539 (6)	
H32	0.0064	1.0560	0.3105	0.065*	
C33	0.06725 (17)	0.9490 (2)	0.25347 (13)	0.0541 (6)	
H33	0.1417	0.9573	0.2788	0.065*	
C34	−0.1843 (2)	1.1296 (3)	0.30349 (17)	0.0733 (8)	
H34A	−0.2519	1.1612	0.3083	0.110*	
H34B	−0.1390	1.1839	0.2905	0.110*	
H34C	−0.1442	1.0979	0.3480	0.110*	
N1	0.11960 (13)	0.81469 (15)	0.17864 (9)	0.0434 (4)	
N2	0.52356 (13)	0.83599 (16)	0.25133 (9)	0.0475 (4)	
O1	0.03516 (15)	0.73016 (17)	0.06980 (10)	0.0708 (5)	
O2	0.29362 (12)	0.66240 (13)	0.13240 (9)	0.0511 (4)	
O3	0.26535 (15)	0.98497 (17)	0.06915 (9)	0.0666 (5)	
O4	0.24443 (15)	1.04707 (14)	0.17193 (10)	0.0616 (5)	
O5	0.55371 (16)	0.84351 (15)	0.10714 (10)	0.0639 (5)	
O6	0.48258 (15)	1.06910 (16)	0.28235 (10)	0.0658 (5)	
O7	−0.21005 (14)	1.05041 (18)	0.25026 (11)	0.0750 (6)	

### Atomic displacement parameters (Å^2^)

**Table:**

	*U*^11^	*U*^22^	*U*^33^	*U*^12^	*U*^13^	*U*^23^
C1	0.0365 (10)	0.0494 (12)	0.0561 (12)	−0.0029 (9)	0.0085 (9)	−0.0051 (10)
C2	0.0376 (10)	0.0395 (11)	0.0607 (12)	−0.0051 (8)	0.0145 (9)	−0.0032 (9)
C3	0.0311 (9)	0.0451 (11)	0.0476 (10)	−0.0006 (8)	0.0102 (8)	0.0025 (9)
C4	0.0309 (8)	0.0410 (11)	0.0427 (10)	−0.0004 (7)	0.0084 (7)	−0.0009 (8)
C5	0.0338 (9)	0.0499 (12)	0.0512 (11)	0.0008 (8)	0.0083 (8)	0.0021 (9)
C6	0.0541 (14)	0.101 (2)	0.0501 (13)	0.0124 (14)	0.0131 (11)	0.0156 (14)
C7	0.0630 (17)	0.109 (3)	0.0643 (17)	−0.0003 (17)	−0.0024 (13)	0.0053 (17)
C8	0.0395 (12)	0.100 (2)	0.0647 (15)	−0.0042 (13)	−0.0028 (11)	0.0059 (15)
C9	0.0309 (9)	0.0485 (12)	0.0448 (10)	−0.0033 (8)	0.0109 (7)	−0.0059 (9)
C10	0.0308 (8)	0.0401 (10)	0.0425 (10)	−0.0036 (7)	0.0077 (7)	−0.0026 (8)
C11	0.0322 (9)	0.0539 (13)	0.0558 (12)	−0.0035 (9)	0.0135 (8)	−0.0120 (10)
C12	0.0351 (10)	0.0510 (13)	0.0590 (13)	−0.0064 (9)	0.0085 (9)	−0.0057 (10)
C13	0.0658 (16)	0.0542 (15)	0.0807 (18)	−0.0097 (12)	0.0118 (14)	−0.0094 (13)
C14	0.089 (2)	0.0647 (18)	0.090 (2)	−0.0250 (16)	0.0134 (18)	0.0087 (17)
C15	0.0711 (18)	0.087 (2)	0.0725 (18)	−0.0257 (16)	0.0138 (14)	0.0177 (17)
C16	0.0476 (12)	0.0776 (18)	0.0566 (13)	−0.0120 (12)	0.0137 (10)	0.0049 (12)
C17	0.0311 (9)	0.0563 (13)	0.0512 (11)	−0.0050 (9)	0.0086 (8)	0.0012 (10)
C18	0.0327 (9)	0.0529 (12)	0.0485 (11)	0.0011 (8)	0.0131 (8)	−0.0045 (9)
C19	0.0340 (9)	0.0434 (11)	0.0495 (11)	−0.0026 (8)	0.0105 (8)	0.0019 (9)
C20	0.088 (2)	0.098 (3)	0.0670 (17)	0.0316 (19)	0.0052 (15)	0.0254 (17)
C21A	0.133 (6)	0.068 (4)	0.107 (5)	0.023 (4)	0.011 (4)	0.022 (3)
C21B	0.080 (6)	0.141 (10)	0.129 (9)	0.019 (6)	0.001 (6)	0.055 (8)
C22	0.0487 (11)	0.0448 (12)	0.0528 (12)	−0.0033 (9)	0.0123 (9)	−0.0031 (10)
C23	0.0611 (14)	0.0724 (18)	0.0618 (14)	−0.0109 (13)	0.0226 (12)	−0.0083 (13)
C24	0.100 (2)	0.118 (3)	0.089 (2)	−0.022 (2)	0.053 (2)	−0.040 (2)
C25	0.130 (3)	0.118 (3)	0.094 (2)	−0.044 (3)	0.054 (2)	−0.059 (2)
C26	0.091 (2)	0.086 (2)	0.094 (2)	−0.0354 (18)	0.0343 (19)	−0.0377 (19)
C27	0.0583 (14)	0.0573 (15)	0.0752 (16)	−0.0134 (12)	0.0231 (12)	−0.0146 (13)
C28	0.0328 (9)	0.0461 (11)	0.0500 (11)	0.0008 (8)	0.0103 (8)	0.0046 (9)
C29	0.0388 (11)	0.0800 (18)	0.0580 (13)	0.0066 (11)	0.0023 (10)	−0.0167 (12)
C30	0.0341 (11)	0.100 (2)	0.0713 (16)	0.0126 (12)	−0.0011 (11)	−0.0178 (15)
C31	0.0366 (10)	0.0601 (14)	0.0599 (13)	0.0074 (9)	0.0137 (9)	0.0025 (11)
C32	0.0401 (11)	0.0583 (14)	0.0641 (14)	−0.0066 (10)	0.0155 (10)	−0.0128 (11)
C33	0.0306 (9)	0.0584 (14)	0.0709 (15)	−0.0067 (9)	0.0098 (9)	−0.0141 (12)
C34	0.0636 (16)	0.082 (2)	0.0797 (19)	0.0160 (14)	0.0285 (14)	−0.0077 (15)
N1	0.0304 (8)	0.0476 (10)	0.0494 (9)	−0.0001 (7)	0.0061 (7)	−0.0033 (8)
N2	0.0293 (8)	0.0623 (12)	0.0488 (10)	0.0019 (7)	0.0073 (7)	0.0034 (8)
O1	0.0556 (10)	0.0786 (13)	0.0656 (11)	0.0073 (9)	−0.0046 (8)	−0.0213 (10)
O2	0.0393 (7)	0.0479 (9)	0.0687 (10)	−0.0074 (6)	0.0192 (7)	−0.0137 (7)
O3	0.0678 (11)	0.0783 (13)	0.0503 (9)	0.0240 (9)	0.0105 (8)	0.0119 (9)
O4	0.0674 (11)	0.0517 (10)	0.0673 (11)	0.0153 (8)	0.0215 (9)	0.0030 (8)
O5	0.0721 (11)	0.0602 (11)	0.0690 (11)	0.0061 (9)	0.0357 (9)	−0.0104 (9)
O6	0.0601 (10)	0.0743 (12)	0.0706 (11)	−0.0127 (9)	0.0307 (9)	−0.0277 (9)
O7	0.0414 (9)	0.0941 (15)	0.0896 (13)	0.0143 (9)	0.0183 (9)	−0.0205 (11)

### Geometric parameters (Å, °)

**Table:**

C1—O1	1.205 (3)	C19—O4	1.201 (3)
C1—N1	1.359 (3)	C19—O3	1.321 (3)
C1—C2	1.522 (3)	C20—C21B	1.489 (11)
C2—O2	1.408 (2)	C20—O3	1.462 (3)
C2—C3	1.554 (3)	C20—C21A	1.4690
C2—H2	0.9800	C20—H20A	0.9700
C3—N1	1.483 (3)	C20—H20B	0.9700
C3—C4	1.521 (3)	C20—H20C	0.9700
C3—H3	0.9800	C20—H20D	0.9700
C4—C10	1.529 (3)	C21A—H21A	0.9600
C4—C5	1.528 (3)	C21A—H21B	0.9600
C4—H4	0.9800	C21A—H21C	0.9600
C5—N2	1.468 (3)	C21B—H21D	0.9600
C5—C6	1.520 (3)	C21B—H21E	0.9600
C5—H5	0.9800	C21B—H21F	0.9600
C6—C7	1.519 (4)	C22—C23	1.376 (3)
C6—H6A	0.9700	C22—O2	1.377 (3)
C6—H6B	0.9700	C22—C27	1.380 (3)
C7—C8	1.497 (4)	C23—C24	1.369 (4)
C7—H7A	0.9700	C23—H23	0.9300
C7—H7B	0.9700	C24—C25	1.378 (5)
C8—N2	1.470 (3)	C24—H24	0.9300
C8—H8A	0.9700	C25—C26	1.368 (5)
C8—H8B	0.9700	C25—H25	0.9300
C9—N2	1.437 (3)	C26—C27	1.364 (4)
C9—C11	1.539 (3)	C26—H26	0.9300
C9—C18	1.544 (3)	C27—H27	0.9300
C9—C10	1.564 (3)	C28—C33	1.363 (3)
C10—C19	1.503 (3)	C28—C29	1.388 (3)
C10—H10	0.9800	C28—N1	1.415 (3)
C11—O6	1.207 (3)	C29—C30	1.366 (4)
C11—C12	1.476 (3)	C29—H29	0.9300
C12—C13	1.385 (4)	C30—C31	1.384 (4)
C12—C17	1.385 (3)	C30—H30	0.9300
C13—C14	1.370 (5)	C31—O7	1.365 (3)
C13—H13	0.9300	C31—C32	1.370 (3)
C14—C15	1.379 (5)	C32—C33	1.385 (3)
C14—H14	0.9300	C32—H32	0.9300
C15—C16	1.367 (4)	C33—H33	0.9300
C15—H15	0.9300	C34—O7	1.405 (4)
C16—C17	1.389 (3)	C34—H34A	0.9600
C16—H16	0.9300	C34—H34B	0.9600
C17—C18	1.471 (3)	C34—H34C	0.9600
C18—O5	1.201 (3)		
			
O1—C1—N1	132.1 (2)	C21B—C20—O3	108.2 (5)
O1—C1—C2	135.9 (2)	C21B—C20—C21A	142.3 (5)
N1—C1—C2	92.01 (17)	O3—C20—C21A	108.85 (17)
O2—C2—C1	117.96 (19)	C21B—C20—H20A	49.7
O2—C2—C3	117.93 (16)	O3—C20—H20A	109.9
C1—C2—C3	86.24 (16)	C21A—C20—H20A	109.9
O2—C2—H2	110.9	C21B—C20—H20B	62.3
C1—C2—H2	110.9	O3—C20—H20B	109.9
C3—C2—H2	110.9	C21A—C20—H20B	109.9
N1—C3—C4	116.81 (17)	H20A—C20—H20B	108.3
N1—C3—C2	86.19 (15)	C21B—C20—H20C	110.1
C4—C3—C2	120.41 (17)	O3—C20—H20C	110.1
N1—C3—H3	110.4	C21A—C20—H20C	49.7
C4—C3—H3	110.4	H20A—C20—H20C	62.9
C2—C3—H3	110.4	H20B—C20—H20C	139.5
C3—C4—C10	116.51 (16)	C21B—C20—H20D	110.1
C3—C4—C5	112.06 (17)	O3—C20—H20D	110.1
C10—C4—C5	103.33 (15)	C21A—C20—H20D	62.2
C3—C4—H4	108.2	H20A—C20—H20D	139.5
C10—C4—H4	108.2	H20B—C20—H20D	50.5
C5—C4—H4	108.2	H20C—C20—H20D	108.4
N2—C5—C6	105.00 (18)	C20—C21A—H21A	109.5
N2—C5—C4	105.16 (17)	C20—C21A—H21B	109.5
C6—C5—C4	118.62 (19)	H21A—C21A—H21B	109.5
N2—C5—H5	109.2	C20—C21A—H21C	109.5
C6—C5—H5	109.2	H21A—C21A—H21C	109.5
C4—C5—H5	109.2	H21B—C21A—H21C	109.5
C5—C6—C7	102.5 (2)	C20—C21B—H21D	109.5
C5—C6—H6A	111.3	C20—C21B—H21E	109.5
C7—C6—H6A	111.3	H21D—C21B—H21E	109.5
C5—C6—H6B	111.3	C20—C21B—H21F	109.5
C7—C6—H6B	111.3	H21D—C21B—H21F	109.5
H6A—C6—H6B	109.2	H21E—C21B—H21F	109.5
C8—C7—C6	104.1 (2)	C23—C22—O2	115.0 (2)
C8—C7—H7A	110.9	C23—C22—C27	120.5 (2)
C6—C7—H7A	110.9	O2—C22—C27	124.6 (2)
C8—C7—H7B	110.9	C22—C23—C24	119.0 (3)
C6—C7—H7B	110.9	C22—C23—H23	120.5
H7A—C7—H7B	109.0	C24—C23—H23	120.5
N2—C8—C7	104.5 (2)	C23—C24—C25	120.5 (3)
N2—C8—H8A	110.9	C23—C24—H24	119.7
C7—C8—H8A	110.9	C25—C24—H24	119.7
N2—C8—H8B	110.9	C26—C25—C24	120.2 (3)
C7—C8—H8B	110.9	C26—C25—H25	119.9
H8A—C8—H8B	108.9	C24—C25—H25	119.9
N2—C9—C11	118.21 (17)	C25—C26—C27	119.8 (3)
N2—C9—C18	113.43 (17)	C25—C26—H26	120.1
C11—C9—C18	102.45 (17)	C27—C26—H26	120.1
N2—C9—C10	102.57 (16)	C26—C27—C22	120.1 (3)
C11—C9—C10	111.24 (16)	C26—C27—H27	120.0
C18—C9—C10	108.94 (16)	C22—C27—H27	120.0
C19—C10—C4	113.86 (16)	C33—C28—C29	119.4 (2)
C19—C10—C9	113.61 (17)	C33—C28—N1	121.92 (18)
C4—C10—C9	102.83 (15)	C29—C28—N1	118.6 (2)
C19—C10—H10	108.8	C30—C29—C28	119.9 (2)
C4—C10—H10	108.8	C30—C29—H29	120.1
C9—C10—H10	108.8	C28—C29—H29	120.1
O6—C11—C12	126.1 (2)	C29—C30—C31	120.8 (2)
O6—C11—C9	125.9 (2)	C29—C30—H30	119.6
C12—C11—C9	107.97 (18)	C31—C30—H30	119.6
C13—C12—C17	120.7 (2)	O7—C31—C32	124.8 (2)
C13—C12—C11	129.3 (2)	O7—C31—C30	116.1 (2)
C17—C12—C11	110.0 (2)	C32—C31—C30	119.2 (2)
C14—C13—C12	117.9 (3)	C31—C32—C33	120.1 (2)
C14—C13—H13	121.0	C31—C32—H32	120.0
C12—C13—H13	121.0	C33—C32—H32	120.0
C13—C14—C15	121.4 (3)	C28—C33—C32	120.7 (2)
C13—C14—H14	119.3	C28—C33—H33	119.7
C15—C14—H14	119.3	C32—C33—H33	119.7
C16—C15—C14	121.3 (3)	O7—C34—H34A	109.5
C16—C15—H15	119.4	O7—C34—H34B	109.5
C14—C15—H15	119.4	H34A—C34—H34B	109.5
C15—C16—C17	117.9 (3)	O7—C34—H34C	109.5
C15—C16—H16	121.0	H34A—C34—H34C	109.5
C17—C16—H16	121.0	H34B—C34—H34C	109.5
C16—C17—C12	120.7 (2)	C1—N1—C28	129.38 (17)
C16—C17—C18	128.8 (2)	C1—N1—C3	95.32 (16)
C12—C17—C18	110.47 (19)	C28—N1—C3	130.41 (17)
O5—C18—C17	127.1 (2)	C9—N2—C5	112.32 (15)
O5—C18—C9	125.1 (2)	C9—N2—C8	120.9 (2)
C17—C18—C9	107.81 (18)	C5—N2—C8	109.91 (18)
O4—C19—O3	124.3 (2)	C22—O2—C2	116.54 (16)
O4—C19—C10	124.9 (2)	C19—O3—C20	116.7 (2)
O3—C19—C10	110.83 (18)	C31—O7—C34	118.0 (2)
			
O1—C1—C2—O2	58.3 (4)	C11—C9—C18—C17	−10.5 (2)
N1—C1—C2—O2	−123.46 (19)	C10—C9—C18—C17	107.36 (19)
O1—C1—C2—C3	178.1 (3)	C4—C10—C19—O4	33.5 (3)
N1—C1—C2—C3	−3.69 (16)	C9—C10—C19—O4	−83.8 (3)
O2—C2—C3—N1	123.2 (2)	C4—C10—C19—O3	−147.20 (18)
C1—C2—C3—N1	3.39 (15)	C9—C10—C19—O3	95.5 (2)
O2—C2—C3—C4	4.2 (3)	O2—C22—C23—C24	−179.9 (3)
C1—C2—C3—C4	−115.6 (2)	C27—C22—C23—C24	−0.5 (4)
N1—C3—C4—C10	−54.3 (2)	C22—C23—C24—C25	0.1 (6)
C2—C3—C4—C10	47.9 (3)	C23—C24—C25—C26	0.0 (7)
N1—C3—C4—C5	−172.95 (17)	C24—C25—C26—C27	0.3 (7)
C2—C3—C4—C5	−70.8 (2)	C25—C26—C27—C22	−0.7 (6)
C3—C4—C5—N2	150.00 (17)	C23—C22—C27—C26	0.8 (4)
C10—C4—C5—N2	23.8 (2)	O2—C22—C27—C26	−179.9 (3)
C3—C4—C5—C6	−93.0 (3)	C33—C28—C29—C30	0.9 (4)
C10—C4—C5—C6	140.7 (2)	N1—C28—C29—C30	177.6 (3)
N2—C5—C6—C7	−29.2 (3)	C28—C29—C30—C31	0.3 (5)
C4—C5—C6—C7	−146.3 (2)	C29—C30—C31—O7	179.4 (3)
C5—C6—C7—C8	37.7 (3)	C29—C30—C31—C32	−1.1 (4)
C6—C7—C8—N2	−31.8 (3)	O7—C31—C32—C33	−179.8 (2)
C3—C4—C10—C19	77.5 (2)	C30—C31—C32—C33	0.8 (4)
C5—C4—C10—C19	−159.12 (17)	C29—C28—C33—C32	−1.2 (4)
C3—C4—C10—C9	−159.09 (17)	N1—C28—C33—C32	−177.8 (2)
C5—C4—C10—C9	−35.8 (2)	C31—C32—C33—C28	0.3 (4)
N2—C9—C10—C19	158.22 (17)	O1—C1—N1—C28	25.5 (4)
C11—C9—C10—C19	30.9 (2)	C2—C1—N1—C28	−152.9 (2)
C18—C9—C10—C19	−81.3 (2)	O1—C1—N1—C3	−177.8 (3)
N2—C9—C10—C4	34.68 (19)	C2—C1—N1—C3	3.88 (17)
C11—C9—C10—C4	−92.64 (19)	C33—C28—N1—C1	179.3 (2)
C18—C9—C10—C4	155.16 (17)	C29—C28—N1—C1	2.7 (4)
N2—C9—C11—O6	−42.0 (3)	C33—C28—N1—C3	30.4 (3)
C18—C9—C11—O6	−167.5 (2)	C29—C28—N1—C3	−146.2 (2)
C10—C9—C11—O6	76.2 (3)	C4—C3—N1—C1	118.5 (2)
N2—C9—C11—C12	136.57 (18)	C2—C3—N1—C1	−3.80 (17)
C18—C9—C11—C12	11.1 (2)	C4—C3—N1—C28	−85.1 (3)
C10—C9—C11—C12	−105.17 (18)	C2—C3—N1—C28	152.6 (2)
O6—C11—C12—C13	−8.3 (4)	C11—C9—N2—C5	102.0 (2)
C9—C11—C12—C13	173.2 (2)	C18—C9—N2—C5	−138.09 (18)
O6—C11—C12—C17	170.6 (2)	C10—C9—N2—C5	−20.8 (2)
C9—C11—C12—C17	−8.0 (2)	C11—C9—N2—C8	−30.4 (3)
C17—C12—C13—C14	−0.8 (4)	C18—C9—N2—C8	89.5 (2)
C11—C12—C13—C14	178.0 (2)	C10—C9—N2—C8	−153.2 (2)
C12—C13—C14—C15	0.0 (5)	C6—C5—N2—C9	−127.4 (2)
C13—C14—C15—C16	0.6 (5)	C4—C5—N2—C9	−1.5 (2)
C14—C15—C16—C17	−0.3 (4)	C6—C5—N2—C8	10.2 (3)
C15—C16—C17—C12	−0.5 (3)	C4—C5—N2—C8	136.1 (2)
C15—C16—C17—C18	−179.1 (2)	C7—C8—N2—C9	146.9 (2)
C13—C12—C17—C16	1.0 (3)	C7—C8—N2—C5	13.5 (3)
C11—C12—C17—C16	−177.95 (19)	C23—C22—O2—C2	176.5 (2)
C13—C12—C17—C18	179.9 (2)	C27—C22—O2—C2	−2.9 (3)
C11—C12—C17—C18	0.9 (2)	C1—C2—O2—C22	−75.8 (2)
C16—C17—C18—O5	4.5 (4)	C3—C2—O2—C22	−177.18 (18)
C12—C17—C18—O5	−174.2 (2)	O4—C19—O3—C20	−1.9 (4)
C16—C17—C18—C9	−174.8 (2)	C10—C19—O3—C20	178.8 (2)
C12—C17—C18—C9	6.4 (2)	C21B—C20—O3—C19	−86.4 (6)
N2—C9—C18—O5	41.5 (3)	C21A—C20—O3—C19	86.9 (2)
C11—C9—C18—O5	170.1 (2)	C32—C31—O7—C34	4.3 (4)
C10—C9—C18—O5	−72.0 (3)	C30—C31—O7—C34	−176.3 (3)
N2—C9—C18—C17	−139.10 (17)		

### Hydrogen-bond geometry (Å, °)

**Table:**

*D*—H···*A*	*D*—H	H···*A*	*D*···*A*	*D*—H···*A*
C2—H2···O6^i^	0.98	2.45	3.151 (3)	129
C26—H26···O1^ii^	0.93	2.50	3.390 (4)	160
C8—H8A···O6	0.97	2.50	3.166 (4)	126
C10—H10···O2	0.98	2.24	2.987 (3)	132
C29—H29···O1	0.93	2.40	3.024 (3)	125

Symmetry codes: (i) −*x*+1/2, *y*−1/2, −*z*+1/2; (ii) −*x*, −*y*+1, −*z*.
